# IL-1 Inhibition in Systemic Juvenile Idiopathic Arthritis

**DOI:** 10.3389/fphar.2016.00467

**Published:** 2016-12-06

**Authors:** Gabriella Giancane, Francesca Minoia, Sergio Davì, Giulia Bracciolini, Alessandro Consolaro, Angelo Ravelli

**Affiliations:** ^1^Pediatric Rheumatology, Istituto Giannina GasliniGenova, Italy; ^2^Pediatria II, Università degli Studi di GenovaGenova, Italy

**Keywords:** systemic juvenile idiopathic arthritis, IL1-inhibitors, anakinra, canakinumab, rilonacept

## Abstract

Systemic juvenile idiopathic arthritis (sJIA) is the form of childhood arthritis whose treatment is most challenging. The demonstration of the prominent involvement of interleukin (IL)-1 in disease pathogenesis has provided the rationale for the treatment with biologic medications that antagonize this cytokine. The three IL-1 blockers that have been tested so far (anakinra, canakinumab, and rilonacept) have all been proven effective and safe, although only canakinumab is currently approved for use in sJIA. The studies on IL-1 inhibition in sJIA published in the past few years suggest that children with fewer affected joints, higher neutrophil count, younger age at disease onset, shorter disease duration, or, possibly, higher ferritin level may respond better to anti-IL-1 treatment. In addition, it has been postulated that use of IL-1 blockade as first-line therapy may take advantage of a “window of opportunity,” in which disease pathophysiology can be altered to prevent the occurrence of chronic arthritis. In this review, we analyze the published literature on IL-1 inhibitors in sJIA and discuss the rationale underlying the use of these medications, the results of therapeutic studies, and the controversial issues.

## Introduction

Systemic juvenile idiopathic arthritis (sJIA) is the most severe form of childhood arthritis and the most difficult to treat. Until recently, sJIA was considered a therapeutic orphan, since the most effective treatment was corticosteroids, whose long-term administration is associated with a wide range of side effects, including an increased risk of vertebral fractures, cataracts, growth retardation, and susceptibility to infection. Traditional disease-modifying anti-rheumatic drugs (DMARDs), such as methotrexate, have limited efficacy for the joint disease and virtually no impact on the systemic features. Poor responses have also been reported with the newer anti-tumor necrosis factor (TNF) agents (Quartier et al., 2003; Horneff et al., 2004; Kimura et al., 2005; Solari et al., 2013), although these medications may be effective in the later afebrile disease phase, characterized by chronic arthritis (Lovell et al., 2008; Giannini et al., 2009). Recently, anti-TNF therapy was found to restore normal levels of vasculoprotective and proangiogenic endothelial progenitor cells in children with JIA (Martini et al., 2015). Several experimental studies have suggested a major pathogenetic role for cytokines such as interleukin (IL)-6 (de Benedetti and Martini, 2005) and, more recently, IL-1 (Pascual et al., 2005). These findings have opened the way to the successful treatment of sJIA with biologic agents that antagonize selectively these cytokines.

In the present review, we provide a brief overview of the main clinical features of sJIA and summarize the recent advances in therapy with IL-1 inhibitors.

## Clinical characteristics of sJIA

**Figure 1:**
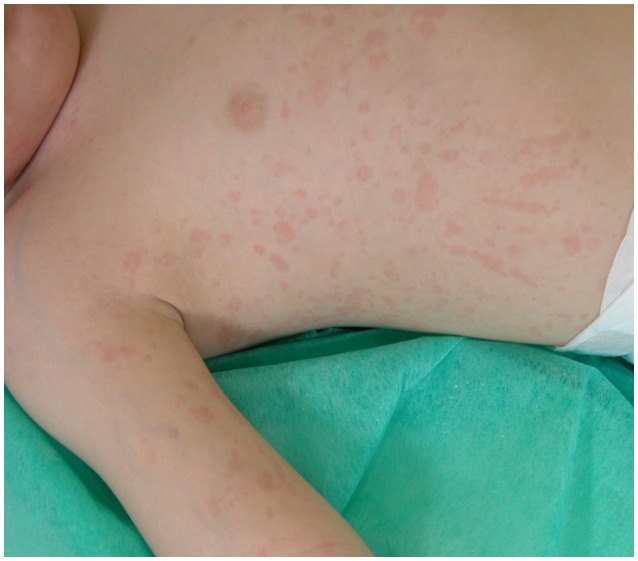
**Salmon-macular rash in systemic juvenile idiopathic arthritis**.

sJIA accounts for 5–15% of all children with chronic arthritis in Europe and North America and is rather distinct from the other forms of JIA, owing to the association of arthritis with a severe systemic illness (Martini, 2012a; De Benedetti and Schneider, 2016). It is considered the childhood-onset equivalent of adult-onset Still's disease. Children with sJIA typically present with a quotidian, high-spiking fever, often accompanied by an erythematous, salmon pink, macular rash, which tends to be migratory and is strikingly evanescent (Figure 1). Myalgias and abdominal pain may be intense during fever peaks. Other systemic manifestations include diffuse lymphoadenopathy, hepatosplenomegaly, and serositis, especially pleuritis and pericarditis. Arthritis is more often symmetrical and polyarticular, but may be absent at onset and develop during the disease course weeks, months, or, rarely, years after the occurrence of extra-articular symptoms. At disease presentation, particularly when arthritis is not yet present, children often require an accurate diagnostic work-up to exclude other potential diagnoses, such as infections and malignancy. Characteristic laboratory features include anemia (usually hypochromic and microcytic), leukocytosis, thrombocytosis, elevated immunoglobulins, increased erythrocyte sedimentation rate (ESR) and C-reactive protein (CRP), and hypoalbuminemia. The International League for Associations of Rheumatology (ILAR) criteria for the classification of sJIA are shown in Table 1.

**Table 1:** **ILAR criteria for sJIA**.

Arthritis with, or preceded by, daily fever of at least 2 weeks' duration that is documented to be quotidian for at least 3 days, and accompanied by one or more of the following:
(1) evanescent, non-fixed, erythematous rash
(2) generalized lymphadenopathy
(3) hepatomegaly or splenomegaly
(4) pericarditis, pleuritis and/or peritonitis
Exclusion criteria
- Psoriasis or a history of psoriasis in patient or first-degree relative
- Arthritis in HLA-B27–positive male >6 years of age
- HLA-B27 associated diseases such as ankylosing spondylitis, enthesitis-related arthritis, sacroiliitis with inflammatory bowel disease, reactive arthritis, or acute anterior uveitis; or history of these in a first-degree relative
- Positive rheumatoid factor test on two occasions ≥ 3 months apart

*Adapted from Petty et al. (2004)*.

It has recently been argued that there are patients not classifiable as sJIA by current criteria who present with the same systemic features seen in classic sJIA, but never develop arthritis (Martini, 2012a). The similarity of clinical manifestations suggest that their illness is closely related to sJIA, despite the absence of arthritis. This subgroup of patients, which nowadays lacks a taxonomic designation, would meet the criteria for adult-onset Still's disease, which do not require the presence of arthritis for diagnosis (Yamaguchi et al., 1992). These considerations have led to propose to include these patients in the sJIA category, and to rename sJIA as Still's disease in order to harmonize the terminology with that of the adult counterpart (Martini, 2012b). A recent analysis of initial clinical features of 136 children with sJIA through a Web-based registry has shown that the ILAR criteria identified only 30% of sJIA patients at disease presentation (Behrens et al., 2008).

**Figure 2:**
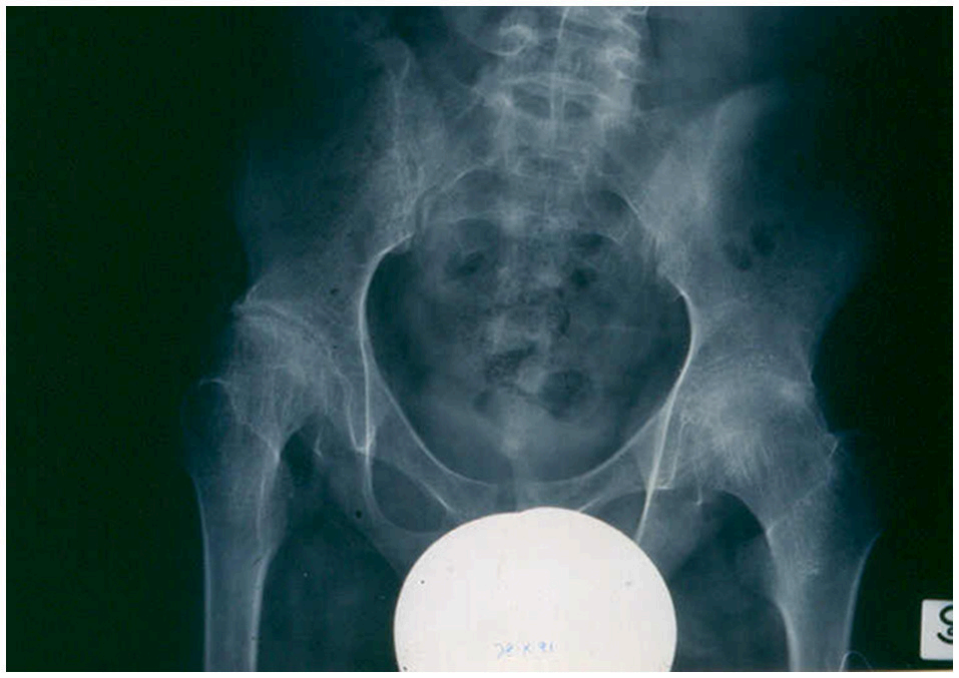
**X-ray showing advanced destructive changes in the hips of a systemic JIA patient**.

The course and prognosis of sJIA are variable (Martini, 2012a; De Benedetti and Schneider, 2016). Around 40% of patients have a good long-term outcome, with a monocyclic course that enters a permanent remission with time. A small proportion of patients have an intermittent course, with relapses followed by periods of quiescence. In the remaining half of the patients, the disease pursues a more severe, persistent disease course. Among this unremitting subset, the sickest children have ongoing systemic symptoms, early destructive polyarthritis (Figure 2), growth failure, and are exposed to the serious side effects of corticosteroids. This particular disease phenotype represents the most disabling of all the different forms of JIA.

**Figure 3:**
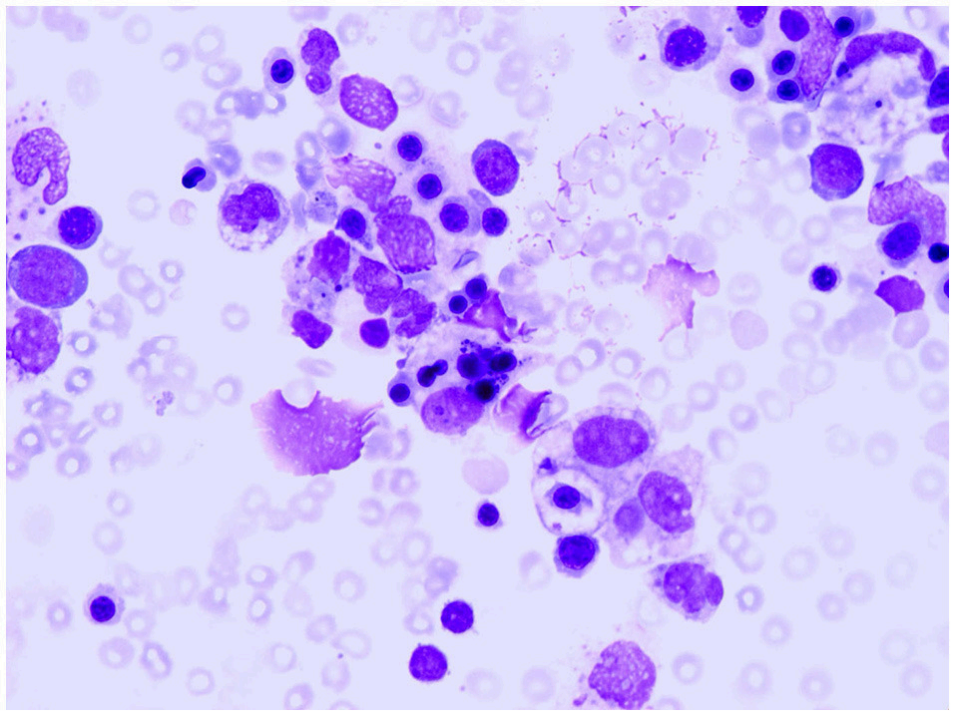
**Bone marrow specimen showing macrophage hemophagocytosis in a patient with systemic arthritis and macrophage activation syndrome**.

Children with sJIA are uniquely susceptible to develop a potentially fatal complication known as macrophage activation syndrome (MAS). MAS is characterized by an overwhelming inflammatory reaction due to an uncontrolled and dysfunctional immune response involving the continued activation and expansion of T lymphocytes and macrophages, with resultant massive hypersecretion of proinflammatory cytokines (Ravelli et al., 2012; Grom et al., 2016). Distinctive clinical features of MAS are high, non-remitting fever, hepatosplenomegaly, generalized lymphadenopathy, central nervous system dysfunction, hemorrhagic manifestations, and, in its most extreme forms, multiorgan failure. Characteristic laboratory abnormalities include pancytopenia, increased levels of ferritin, liver enzymes, lactate dehydrogenase, triglycerides, D-dimers, and soluble IL-2 receptor a (also known as soluble CD25), and decreased fibrinogen levels. A characteristic histopathologic feature of MAS is the accumulation of well-differentiated macrophages exhibiting hemophagocytic activity in bone marrow biopsy specimens or aspirates (Figure 3; Ravelli, 2002). Although ~10% of sJIA patients develop overt MAS, up to 30% of children have evidence of subclinical MAS (Behrens et al., 2007; Bleesing et al., 2007). MAS can result in progressive multi-organ failure and eventually a fatal outcome if unrecognized. Recent studies indicate a mortality rate of 8% (Minoia et al., 2014, 2015). In 2016, classification criteria for MAS complicating sJIA have been published (Table 2) (Ravelli et al., 2015, 2016).

**Table 2:** **2006 classification criteria of MAS**.

A febrile patient with known or suspected systemic juvenile idiopathic arthritis is classified as having macrophage activation syndrome if the following criteria are met:
Ferritin > 684 ng/ml and any two of the following:
Platelet count ≤ 181 × 10^9^/liter
Aspartate aminotransferase > 48 units/liter
Triglycerides > 156 mg/dl
Fibrinogen ≤ 360 mg/dl

*Adapted from Ravelli et al. (2015, 2016)*.

## Interleukin-1 inhibitors in the management of sJIA

### Anakinra

The first observation of successful treatment of sJIA with IL-1 inhibition dates back to 2004, when a remarkable response to the recombinant interleukin (IL)-1 receptor antagonist anakinra in two patients with severe and refractory disease manifestations was described (Verbsky and White, 2004).

In a landmark study published in 2005, Pascual et al. (2005) reported that the administration of anakinra to 9 children with active sJIA refractory to other therapies led to striking improvement in clinical symptoms and inflammatory markers. Seven patients achieved complete remission and the other 2 patients had a partial response. The rationale for the treatment was provided by the demonstration that patients' serum induced the transcription of innate immunity genes, included those of IL-1α and IL-1β, in healthy peripheral-blood mononuclear cells, and that patients' peripheral-blood mononuclear cells produced an excess of IL-1β upon activation.

A less impressive effectiveness was seen in a French multicenter, randomized, double-blind, placebo-controlled trial (ANAJIS trial), whose primary outcome was the achievement of an American College of Rheumatology Pediatric (ACR Pedi) 30 response at 1 month. At treatment endpoint, 8 of 12 patients (67%) in the anakinra group and only 1 of 12 patients (8%) in the placebo group were responders (*p* = 0.003). However, no patient in both groups achieved a more robust improvement (i.e., a modified ACR Pedi 100 response). Furthermore, loss of response was observed in most patients over time. The authors attributed the frequent lack of sustained efficacy to the presence of severe polyarthritis and the absence of fever in most patients at enrolment, to the possible insufficient dosage in younger patients, and to the study design, which precluded the concomitant use of DMARDs and allowed early tapering of corticosteroids. Notably, a *de novo* type I interferon signature, which is not a feature of untreated sJIA, was induced in the majority of anakinra-treated patients, regardless of clinical response (Quartier et al., 2011).

That anakinra could be less effective on arthritis symptoms than on systemic and laboratory features of inflammation was highlighted in a retrospective study by Gattorno et al. (2008). By examining the pattern of response to anakinra in 22 children with sJIA, they identified two groups of patients: one group exhibited a dramatic response, with rapid improvement of arthritis and normalization of the CRP within the first week of treatment; the other group had no response or experienced only transient improvement of joint disease and CRP. The only difference between responders and non-responders or incomplete responders was a lesser extension of arthritis and an increased absolute neutrophil count in the former group. *In vitro* secretion of IL-1β and IL-18 by patient monocytes was not increased and was independent of both treatment outcome and disease activity. Other case series published around the same time also showed remarkable benefit among many, but not all, users of anakinra (Lequerré et al., 2008; Ohlsson et al., 2008; Zeft et al., 2009).

Recent observations suggest that initiation of anakinra early in the disease course may improve outcome. A multicenter retrospective cohort study of 46 patients who had received anakinra as part of initial corticosteroid-sparing regimen showed that around 60%, including 8 of 10 receiving anakinra monotherapy, attained a complete response without escalation of therapy. Almost all patients had rapid improvements in fever and rash, whereas a slower response of arthritis to treatment was seen, with persistently active synovitis in 39% of patients at 1 month, 27% of patients at 3 months, and 15% of patients at 6 months. Inflammatory markers normalized in most patients within 1 month. Evidence that early intervention with anakinra could prevent the development of persistent synovitis was obtained for 91% of 35 patients followed up for at least 6 months. Disease characteristics and treatment were similar in patients with partial or absent response and patients with complete response, except that that the former patients were markedly younger at disease onset (median age 5.2 years vs. 10.2 years; *P* = 0.004). Notably, however, the median peak ferritin level was higher in complete responders than in partial or non-responders (3008 vs. 1329 ng/ml). Although the difference was not significant, perhaps owing to the small size of the study population, this observation suggests that patients with more prominent activation of the monocyte/macrophage system are more responsive to IL-1 inhibition (Nigrovic et al., 2011).

Vastert et al. (2014) conducted the first prospective study of the use of an IL-1 antagonist as first-line therapy in sJIA. They started anakinra in 20 patients with new-onset sJIA who were corticosteroid-naïve. At 3 months, 85% of patients achieved an adapted ACR Pedi 90 response or had inactive disease; 75% of patients achieved this response while receiving anakinra monotherapy. In the majority of responding patients (73%), treatment could be stopped within 1 year, with remission being preserved during follow-up. However, in around one third of patients, concomitant therapy was required for maintenance of clinical response. IL-18 as well as the myeloid-related proteins (MRP) S100A12 and S100A8/9 were found to be potential biomarkers for guiding the strategy of stopping treatment with IL-1 inhibitors.

A recent single-center experience with anakinra therapy in 25 patients with sJIA showed that 56% of patients attained inactive disease. The only baseline variable significantly associated with response was the time interval disease onset and treatment start, with earlier treatment being associated with better outcome. Once more, however, the median ferritin level tended to be higher in patients who reached inactive disease than in those who did not (1506 vs. 360 ng/ml). Importantly, the comparison of the dose administered with the ideal dose of anakinra in each individual patient did not show any relation with therapeutic response (Pardeo et al., 2015).

In spite of the demonstrations of its effectiveness, anakinra is not currently registered for the treatment of sJIA.

### Canakinumab

A preliminary phase II, multicenter, open-label study evaluated dosing, efficacy, and safety of the fully human anti-IL-1β antibody canakinumab in 23 children with sJIA and active systemic features. This analysis showed that the administration of 4 mg/kg was associated with rapid and sustained improvement in clinical response and enabled reduction or discontinuation of corticosteroids. In keeping with the findings of the aforementioned study by Gattorno et al. (2008), responders to canakinumab had fewer active joints and a higher white blood cell count at baseline than did non-responders (Ruperto et al., 2010).

The results of this pilot study provided the basis for performing two double-blind placebo-controlled trials of canakinumab in a larger population of sJIA patients with active systemic features (Ruperto et al., 2012). In the first trial, 84% of patients receiving a single injection of canakinumab compared with only 10% of those receiving placebo achieved an ACR Pediatric 30 response with no fever (*p* < 0.001). The frequency of inactive disease in the canakinumab group was as high as 33% after only 15 days. In the second trial, conducted with a withdrawal design, 73% of the patients demonstrated at least an ACR Pediatric 50 response and no fever and 31% had inactive disease at the end of the open-label phase, after a median of 113 days. In the randomized withdrawal phase, the frequency of flare was markedly lower in the canakinumab group than in the placebo group (74% of patients in the canakinumab group had no flare, vs. 25% in the placebo group; *P* = 0.003). At the end of the withdrawal phase, 62% of canakinumab-treated patients and 34% of patients in the placebo group had inactive disease. The average corticosteroid dose was reduced from 0.34 to 0.05 mg/kg/day and corticosteroids were discontinued in 33% of patients. Medication safety was overall good, although infections were more frequent with canakinumab than with placebo and 7 patients had MAS.

Canakinumab has been approved for the treatment for the treatment of active sJIA in children aged 2 years and older both in Europe and in the US.

### Rilonacept

The efficacy and safety of the anti-IL-1 soluble decoy receptor protein, rilonacept, were evaluated in a pilot 3-phase trial consisting in a 23 months of open-label treatment preceded by a 4 week, double-blind, placebo-controlled phase. Although no significant differences in efficacy were observed between the rilonacept- and placebo-treated patients during the initial double-blind phase, fever and rash completely resolved by month 3 in all patients during the open-label treatment period and did not recur. The adapted ACR Pedi 30, 50, and 70 response rates at 3 months were 78.3, 60.9, and 34.8%, respectively, and were generally maintained over the study duration. In addition to declines in high-sensitivity CRP, reductions were seen in the levels of MRP-8/MRP-14 and D-dimer. In 22 of 23 patients, prednisone was tapered or discontinued. Treatment was not associated with serious adverse events (Lovell et al., 2013).

A larger 24 week randomized trial of the same agent in 71 children with active arthritis in ≥2 joints, which incorporated a 4 week double-blind placebo phase, found a shorter time to response in the rilonacept arm than in the placebo arm (*P* = 0.007). In a secondary analysis, 57% of the patients in the rilonacept arm had a response at week 4 compared with 27% of the patients in the placebo arm (*P* = 0.016). No statistically significant association was observed between a poorer response at week 4 and absence of systemic manifestations or longer disease duration. However, the median disease duration tended to shorter among patients who responded at week 4 compared to those who did not. The medication was generally well-tolerated (Ilowite et al., 2014).

Thus far, rilonacept has not been approved for use in children with sJIA.

## Open issues and future outlook

The advent of biologic agents that specifically inhibit IL-1 has dramatically improved clinical outcomes for many children with sJIA and confirmed the pathogenic role of this cytokine in disease processes. The demonstration of the prominent involvement of IL-1, together with the lack of HLA associations and autoantibodies and the strong implication of cells of the innate immune system, has led to the suggestion that sJIA is a distinct disease entity, with more similarities with autoinflammatory syndromes than with classic autoimmune diseases (Masters et al., 2009; Vastert et al., 2009; Mellins et al., 2011; Martini, 2012b).

However, not all patients respond to IL-1 blockade (Gattorno et al., 2008; Lequerré et al., 2008; Swart et al., 2010; Quartier et al., 2011). The varying susceptibility to anti-IL-1 therapy may be explained by the heterogeneity of sJIA. The aforementioned analysis of the pattern of response to anakinra identified two patient subsets, one with dramatic response, similar to that observed in cryopirin-associated autoinflammatory syndromes, and the other resistant or with an intermediate response. Patients responding to anti-IL-1 therapy had fewer affected joints and a higher neutrophil count (Gattorno et al., 2008). This observation has led to postulate that the group with bright response represents a separate entity in which autoinflammatory mechanisms play the leading pathogenetic role, whereas the group with more severe arthritis may also have autoimmune components (Martini, 2012a). Other investigators have found evidence that anti-IL-1 treatment may be more effective for systemic features than for articular manifestations of the disease (Lovell et al., 2013). However, in the canakinumab study, the response to treatment of children with polyarthritis was similar to those without polyarthritis. A differential therapeutic response based on the presence or absence of systemic features could not be evaluated in this trial because all children enrolled had ongoing fever (Ruperto et al., 2012).

The heterogeneous nature of sJIA has been further highlighted by Shimizu and co-workers (Shimizu et al., 2013), who delineated two distinct sJIA patient subsets based on their serum IL-6 and IL-18 levels: an IL-6 dominant and an IL-18 dominant. The IL-6-dominant subset had a more severe polyarthritis and higher serum levels of matrix metalloproteinase (MMP-3), whereas the IL-18-dominant subset was more prone to develop MAS. Whether the differences in the predominant cytokine expression or in the susceptibility to anti-cytokine therapies dissect the spectrum of systemic JIA into clinically or pathogenetically distinct disease entities, remains to be established.

As noticed above, the tendency for ferritin level to be higher in responders to anakinra in some series suggests that patients with more pronounced activation of the monocyte system, which may predispose them to the progression to overt MAS, may be more susceptible to benefit from IL-1 inhibition. This hypothesis is in keeping with the recent reports of the effectiveness of anakinra in cases of MAS refractory to conventional therapies (Ravelli et al., 2012).

Another explanation for the inconsistent effectiveness of IL-1 inhibition could be the timing of therapy. Nearly all patients included in earlier open studies and in randomized clinical trials had long-standing disease and were still receiving systemic corticosteroids when treatment with IL-1 blocking agents was initiated. These characteristics may account for the partial or absent responses seen in a significant minority of patients. More favorable outcomes were obtained with the use of IL-blockade as first-line therapy, particularly in patients with new-onset disease and not yet exposed to corticosteroids or other DMARDs (Nigrovic et al., 2011; Vastert et al., 2014). Many patients achieved inactive disease rapidly and were able to stop anti-IL-1 therapy within 1 year, with sustained remission during follow-up (Vastert et al., 2014). Of equal importance was the observation of a significant reduction in the proportion of children who developed the chronic polyarthritis manifestation of their disease (Nigrovic et al., 2011).

The differential clinical responses in early vs. late disease, coupled with data from animal models, have led to theorize a biphasic model of sJIA, in which the disease begins with a highly inflammatory febrile phase that, in more than half of the patients, converts over time to an afebrile phase characterized by chronic arthritis. The predominance of innate immune mechanisms in the early systemic stage, as opposed to the involvement of autoreactive T-cells in the later induction of chronic arthritis, would explain why antagonism of IL-1 in new-onset disease is associated with better outcomes than those observed when this therapy is initiated later in the disease course. Thus, early treatment with IL-1 inhibitors may take advantage of this “window of opportunity,” in which disease pathophysiology can be altered to avoid the occurrence of chronic arthritis (Nigrovic, 2014).

However, although this hypothesis is logical and attractive, its clinical background should be regarded in the light of some caveats. Because around 40% of patients with sJIA have a monocyclic course with spontaneous remission, results of open studies on patients with early disease may be biased toward patients destined to a milder course. Conversely, most patients enrolled in clinical trials had already had years of disease and, therefore, are unlikely to include patients with a monophasic course. In addition, the majority of these patients had proven refractory to other therapies. Thus, the observed different efficacy of IL-1 blockade between early and established sJIA could simply reflect the fact that the latter patient subset may be more challenging to treat. Nevertheless, although the hypothesis of a window of opportunity is far from proven, it should become the focus of further research into the pathophysiology of sJIA and, possibly, the objective of further multicenter trials in large populations, ideally combined with biomarker analyses.

Since there are now three IL-1 inhibitors on the market, the question arises about which of them is preferable. Not only they differ in the molecular structure, but the mechanism of action is slightly different: anakinra blocks both IL-1α and IL-1β, canakinumab inhibits only IL-1β, and rilonacept binds IL-1α, IL-1β, and IL-1 receptor antagonist. However, it is still unknown whether the different biding properties translate into differential clinical effects (Beukelman, 2014). Anakinra has been the first agent tested and is, thus, the one for which more experience has been gained (although it is not registered for the treatment of sJIA). It has a short half-life of 4–6 h, which is advantageous for handling a major adverse event and provides a greater flexibility for the management of a medical emergency like MAS. However, the need of daily subcutaneous administrations, which are often associated with injection site reactions, may make it difficult to conduct therapy over long-term, particularly in younger children (Lequerré et al., 2008; Quartier et al., 2011). The longer half-life of canakinumab, which enables its administration every 4 weeks, together with its blockage limited to IL-1β, makes this medication potentially better accepted and tolerated. Rilonacept could offer an alternative with its circulating half-life of 8.6 days, in contrast to the long biologic activity of canakinumab (236 days), which could be a disadvantage in the setting of a serious toxic effect. Importantly, significant responses to canakinumab and rilonacept were seen in many patients who had previously been treated with anakinra, which suggests that failure of one anti-IL-1 therapy does not necessarily preclude use of another (Lovell et al., 2013). Last but not least, the issue of cost may have a major impact on the choice of a particular molecule. The dosage, route of administration and half-life of the IL-1 inhibitors used in the management of sJIA is reported in Table 3.

**Table 3:** **Characteristics of the IL-1 inhibitors used for the treatment of sJIA**.

	**Dosage**	**Route of administration**	**Half-life**
Anakinra	1–4 mg/kg/day	Subcutaneous	4–6 h
Canakinumab	≥2 years: 4 mg/kg/dose q 4 weeks	Subcutaneous	23–26 days
	Maximum dose: 300 mg		
Rilonacept	Starting dose 4.4 mg/kg, then 2.2 mg/kg/week	Subcutaneous	1 week
	Maximum loading dose: 320 mg		
	Maximum weekly dose: 160 mg/week		

Overall, all anti-IL-1 agents have proven safe and well-tolerated. However, concerns have been raised regarding the risk of infection, neutropenia, and liver dysfunction (Canna et al., 2009; Sandborg and Mellins, 2012; Buckland, 2013). Furthermore, several instances of MAS during treatment with IL-1 inhibitors, some of which with a fatal outcome, have been seen in clinical practice, randomized controlled trials, and post-marketing experience (Grom and Mellins, 2010; Ruperto et al., 2012). The same phenomenon was reported during treatment with the IL-6 blocker tocilizumab (De Benedetti et al., 2012; Yokota et al., 2012). As discussed elsewhere, the occurrence of MAS during treatment with medications that inhibit proinflammatory cytokines implicated in its pathogenesis is a paradoxical phenomenon. Possible explanations include the increased rate of infections (which, in turn, may trigger MAS) associated with biologic therapies or the induction of an imbalance between up- and down-regulation of the various molecules that are part of the cytokine network (Ravelli et al., 2012; Minoia et al., 2014). However, these episodes of MAS often abated after increasing the dose of biologic medications, which suggests a lack of causality and a real associative relationship in only a few instances.

Treatment targeting another cytokine implicated in the pathogenesis of sJIA, such as the IL-6 blocker tocilizumab, has also demonstrated efficacy in clinical trials (De Benedetti et al., 2012; Yokota et al., 2012). So far, however, there are no clinical data that allow either to compare the effectiveness and safety of IL-1 and IL-6 antagonists or to establish their relative indications in sJIA.

Additional investigations are needed to define the exact role of the currently available agents in the management of sJIA. Future studies will likely optimize the care of children with sJIA and further elucidate the disease pathogenesis.

## Author contributions

All authors listed, have made substantial, direct and intellectual contribution to the work, and approved it for publication.

### Conflict of interest statement

The authors declare that the research was conducted in the absence of any commercial or financial relationships that could be construed as a potential conflict of interest.
